# Titanium germanium anti­monide, TiGeSb

**DOI:** 10.1107/S1600536809031559

**Published:** 2009-08-15

**Authors:** Robert Lam, Arthur Mar

**Affiliations:** a Department of Chemistry, University of Alberta, Edmonton, AB, Canada T6G 2G2

## Abstract

TiGeSb adopts the PbFCl- or ZrSiS-type structure, with Ti atoms (4*mm* symmetry) centred within monocapped square anti­prisms generated by the stacking of denser square nets of Ge atoms (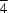 *m*2 symmetry) alternating with less dense square nets of Sb atoms (4*mm* symmetry).

## Related literature

For PbFCl- or ZrSiS-type structures, see: Tremel & Hoffmann (1987 ▶). For a previous report on TiGeSb, see: Dashjav & Kleinke (2002 ▶). The Ti—Ge—Sb phase diagram at 670 K was reported by Kozlov & Pavlyuk (2004 ▶). For the related ZrGeSb, see: Lam & Mar (1997 ▶). For background to solid solutions in this class of compounds, see: Soheilnia *et al.* (2003 ▶); Kozlov & Pavlyuk (2004 ▶). Metallic radii were taken from Pauling (1960 ▶).

## Experimental

### 

#### Crystal data

TiGeSb
*M* *_r_* = 242.24
Tetragonal, 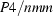

*a* = 3.7022 (5) Å
*c* = 8.2137 (12) Å
*V* = 112.58 (3) Å^3^
*Z* = 2
Mo *K*α radiation
μ = 28.18 mm^−1^
*T* = 295 K
0.12 × 0.11 × 0.01 mm

#### Data collection

Enraf–Nonius CAD-4 diffractometer
Absorption correction: numerical (*SHELXTL*; Sheldrick, 2008 ▶) *T*_min_ = 0.117, *T*_max_ = 0.718
1906 measured reflections
181 independent reflections
178 reflections with *I* > 2σ(*I*)
*R*_int_ = 0.125
3 standard reflections frequency: 120 min intensity decay: none

#### Refinement

*R*[*F* ^2^ > 2σ(*F* ^2^)] = 0.032
*wR*(*F* ^2^) = 0.081
*S* = 1.17
181 reflections
10 parameters
Δρ_max_ = 1.95 e Å^−3^
Δρ_min_ = −2.53 e Å^−3^

### 

Data collection: *CAD-4-PC* (Enraf–Nonius, 1993 ▶); cell refinement: *CAD-4-PC*; data reduction: *XCAD4* (Harms & Wocadlo, 1995 ▶); program(s) used to solve structure: *SHELXTL* (Sheldrick, 2008 ▶); program(s) used to refine structure: *SHELXTL*; molecular graphics: *ATOMS* (Dowty, 1999 ▶); software used to prepare material for publication: *SHELXTL*.

## Supplementary Material

Crystal structure: contains datablocks global, I. DOI: 10.1107/S1600536809031559/wm2247sup1.cif

Structure factors: contains datablocks I. DOI: 10.1107/S1600536809031559/wm2247Isup2.hkl

Additional supplementary materials:  crystallographic information; 3D view; checkCIF report

## Figures and Tables

**Table 1:** Selected bond lengths (Å)

Ti—Ge	2.7570 (10)
Ti—Sb^i^	2.8452 (7)
Ti—Sb	3.0129 (14)
Ge—Ge^ii^	2.6179 (4)

Symmetry codes: (i) 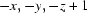; (ii) 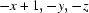.

**Fig. 1.**
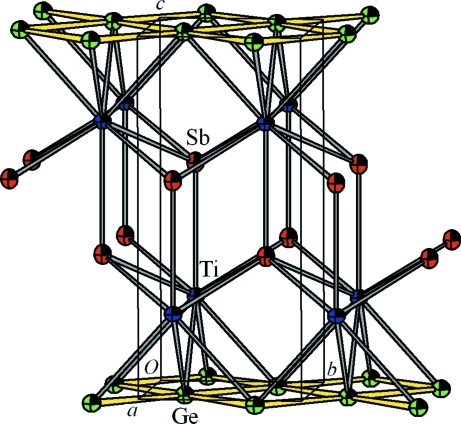
Projection of the TiGeSb structure approximately along the a axis. Displacement ellipsoids are drawn at the 90% probability level.

## supplementary crystallographic information

### Comment

After a report of the ternary antimonide ZrGeSb (Lam & Mar, 1997), the corresponding Ti and Hf analogues were later described in a conference proceeding, but full crystallographic details have not been forthcoming (Dashjav & Kleinke, 2002). The complete structure of TiGeSb, which is absent in the Ti—Ge—Sb phase diagram at 670 K (Kozlov & Pavlyuk, 2004) but was prepared here at 1273 K, is presented. Common to many equiatomic compounds of the formulation *MAB* (*M* = large transition-metal atom; *A*, *B* = main group atoms), TiGeSb adopts the PbFCl- or ZrSiS-type structure, among other names (Tremel & Hoffmann, 1987). Square nets of each type of atom, with the Ge net being twice as dense as the other two, are stacked along the *c* axis (Fig. 1). The Zr atoms are nine-coordinate, centred within monocapped square antiprisms. The Ge–Ge distances are 0.13 Å longer than the sum of the Pauling metallic radii (2.48 Å; Pauling, 1960), indicative of weak polyanionic bonding. The solid solutions ZrGe*_x_*Sb_1-_*_x_* and HfGe*_x_*Sb_1-_*_x_* (up to *x* = 0.2) form related orthorhombic PbCl_2_-type structures (Soheilnia *et al.*, 2003), whereas TiGe*_x_*Sb_1-_*_x_* adopts a NiAs-type structure (Kozlov & Pavlyuk, 2004).

### Experimental

A 0.25 g mixture of Ti (99.98%, Cerac), Ge (99.999%, Cerac), and Sb (99.995%, Aldrich) powders in a 1:1:3 molar ratio was placed in an evacuated fused-silica tube. The tube was heated at 873 K for 2 d and 1273 K for 2 d. Silver plate-shaped crystals were obtained, which were found by semiquantitative energy-dispersive X-ray (EDX) analysis to have a composition (at%) of 32 (2)% Ti, 35 (2)% Ge, and 33 (2)% Sb, in good agreement with the formula TiGeSb.

### Refinement

Analysis of Weissenberg photographs on a plate-shaped crystal, subsequently transferred to the four-circle diffractometer, established Laue symmetry 4/*mmm* and provided approximate cell parameters of *a* = 3.71 Å and *c* = 8.22 Å. In the final Fourier map based on origin choice 2 of space group *P*4/*nmm* the maximum peak and deepest hole are located 0.67 Å and 0.02 Å, respectively, from the Sb atom.

### Crystal data

**Table:**

TiGeSb	*D*_x_ = 7.146 Mg m^−^^3^
*M**_r_* = 242.24	Mo *K*α radiation, λ = 0.71073 Å
Tetragonal, *P*4/*n**m**m*	Cell parameters from 24 reflections
Hall symbol: -P 4a 2a	θ = 11.0–23.3°
*a* = 3.7022 (5) Å	µ = 28.18 mm^−^^1^
*c* = 8.2137 (12) Å	*T* = 295 K
*V* = 112.58 (3) Å^3^	Plate, silver
*Z* = 2	0.12 × 0.11 × 0.01 mm
*F*(000) = 210	

### Data collection

**Table:**

Enraf–Nonius CAD-4 diffractometer	178 reflections with *I* > 2σ(*I*)
Radiation source: fine-focus sealed tube	*R*_int_ = 0.125
graphite	θ_max_ = 34.8°, θ_min_ = 2.5°
θ/2θ scans	*h* = −5→5
Absorption correction: numerical (*SHELXTL*; Sheldrick, 2008)	*k* = −5→5
*T*_min_ = 0.117, *T*_max_ = 0.718	*l* = −13→13
1906 measured reflections	3 standard reflections every 120 min
181 independent reflections	intensity decay: none

### Refinement

**Table:**

Refinement on *F*^2^	Primary atom site location: structure-invariant direct methods
Least-squares matrix: full	Secondary atom site location: difference Fourier map
*R*[*F*^2^ > 2σ(*F*^2^)] = 0.032	*w* = 1/[σ^2^(*F*_o_^2^) + (0.044*P*)^2^ + 0.3679*P*] where *P* = (*F*_o_^2^ + 2*F*_c_^2^)/3
*wR*(*F*^2^) = 0.081	(Δ/σ)_max_ < 0.001
*S* = 1.17	Δρ_max_ = 1.95 e Å^−^^3^
181 reflections	Δρ_min_ = −2.53 e Å^−^^3^
10 parameters	Extinction correction: *SHELXTL* (Sheldrick, 2008), Fc^*^=kFc[1+0.001xFc^2^λ^3^/sin(2θ)]^-1/4^
0 restraints	Extinction coefficient: 0.038 (9)

### Special details

**Table:**

Geometry. All e.s.d.'s (except the e.s.d. in the dihedral angle between two l.s. planes) are estimated using the full covariance matrix. The cell e.s.d.'s are taken into account individually in the estimation of e.s.d.'s in distances, angles and torsion angles; correlations between e.s.d.'s in cell parameters are only used when they are defined by crystal symmetry. An approximate (isotropic) treatment of cell e.s.d.'s is used for estimating e.s.d.'s involving l.s. planes.
Refinement. Refinement of *F*^2^ against ALL reflections. The weighted *R*-factor *wR* and goodness of fit *S* are based on *F*^2^, conventional *R*-factors *R* are based on *F*, with *F* set to zero for negative *F*^2^. The threshold expression of *F*^2^ > σ(*F*^2^) is used only for calculating *R*-factors(gt) *etc*. and is not relevant to the choice of reflections for refinement. *R*-factors based on *F*^2^ are statistically about twice as large as those based on *F*, and *R*- factors based on ALL data will be even larger.

### Fractional atomic coordinates and isotropic or equivalent isotropic displacement parameters (Å^2^)

**Table:**

	*x*	*y*	*z*	*U*_iso_*/*U*_eq_	
Ti	0.2500	0.2500	0.24875 (16)	0.0054 (3)	
Ge	0.7500	0.2500	0.0000	0.0063 (3)	
Sb	0.2500	0.2500	0.61556 (6)	0.0063 (3)	

### Atomic displacement parameters (Å^2^)

**Table:**

	*U*^11^	*U*^22^	*U*^33^	*U*^12^	*U*^13^	*U*^23^
Ti	0.0060 (4)	0.0060 (4)	0.0043 (6)	0.000	0.000	0.000
Ge	0.0065 (4)	0.0065 (4)	0.0059 (4)	0.000	0.000	0.000
Sb	0.0056 (3)	0.0056 (3)	0.0076 (4)	0.000	0.000	0.000

### Geometric parameters (Å, °)

**Table:**

Ti—Ge^i^	2.7570 (10)	Ge—Ge^ix^	2.6179 (3)
Ti—Ge^ii^	2.7570 (10)	Ge—Ti^i^	2.7570 (10)
Ti—Ge^iii^	2.7570 (10)	Ge—Ti^x^	2.7570 (10)
Ti—Ge	2.7570 (10)	Ge—Ti^iii^	2.7570 (10)
Ti—Sb^iv^	2.8452 (7)	Sb—Ti^iv^	2.8452 (7)
Ti—Sb^v^	2.8452 (7)	Sb—Ti^v^	2.8452 (7)
Ti—Sb^vi^	2.8452 (7)	Sb—Ti^vi^	2.8452 (7)
Ti—Sb^vii^	2.8452 (7)	Sb—Ti^vii^	2.8452 (7)
Ti—Sb	3.0129 (14)	Sb—Sb^v^	3.2337 (7)
Ge—Ge^viii^	2.6179 (4)	Sb—Sb^iv^	3.2337 (7)
Ge—Ge^i^	2.6179 (4)	Sb—Sb^vii^	3.2337 (7)
Ge—Ge^iii^	2.6179 (4)	Sb—Sb^vi^	3.2337 (7)
			
Ge^i^—Ti—Ge^ii^	56.69 (2)	Ti^i^—Ge—Ti^x^	123.31 (2)
Ge^i^—Ti—Ge^iii^	84.35 (4)	Ge^viii^—Ge—Ti	118.344 (11)
Ge^ii^—Ti—Ge^iii^	56.69 (2)	Ge^i^—Ge—Ti	61.656 (11)
Ge^i^—Ti—Ge	56.69 (2)	Ge^iii^—Ge—Ti	61.656 (11)
Ge^ii^—Ti—Ge	84.35 (4)	Ge^ix^—Ge—Ti	118.344 (11)
Ge^iii^—Ti—Ge	56.69 (2)	Ti^i^—Ge—Ti	123.31 (2)
Ge^i^—Ti—Sb^iv^	136.65 (2)	Ti^x^—Ge—Ti	84.35 (4)
Ge^ii^—Ti—Sb^iv^	136.65 (2)	Ge^viii^—Ge—Ti^iii^	61.656 (11)
Ge^iii^—Ti—Sb^iv^	81.574 (14)	Ge^i^—Ge—Ti^iii^	118.344 (11)
Ge—Ti—Sb^iv^	81.574 (14)	Ge^iii^—Ge—Ti^iii^	61.656 (11)
Ge^i^—Ti—Sb^v^	81.574 (14)	Ge^ix^—Ge—Ti^iii^	118.344 (11)
Ge^ii^—Ti—Sb^v^	81.574 (14)	Ti^i^—Ge—Ti^iii^	84.35 (4)
Ge^iii^—Ti—Sb^v^	136.65 (2)	Ti^x^—Ge—Ti^iii^	123.31 (2)
Ge—Ti—Sb^v^	136.65 (2)	Ti—Ge—Ti^iii^	123.31 (2)
Sb^iv^—Ti—Sb^v^	133.88 (5)	Ti^iv^—Sb—Ti^v^	133.88 (5)
Ge^i^—Ti—Sb^vi^	136.65 (2)	Ti^iv^—Sb—Ti^vi^	81.17 (2)
Ge^ii^—Ti—Sb^vi^	81.574 (14)	Ti^v^—Sb—Ti^vi^	81.17 (2)
Ge^iii^—Ti—Sb^vi^	81.574 (14)	Ti^iv^—Sb—Ti^vii^	81.17 (2)
Ge—Ti—Sb^vi^	136.65 (2)	Ti^v^—Sb—Ti^vii^	81.17 (2)
Sb^iv^—Ti—Sb^vi^	81.17 (2)	Ti^vi^—Sb—Ti^vii^	133.88 (5)
Sb^v^—Ti—Sb^vi^	81.17 (2)	Ti^iv^—Sb—Ti	113.06 (3)
Ge^i^—Ti—Sb^vii^	81.574 (14)	Ti^v^—Sb—Ti	113.06 (3)
Ge^ii^—Ti—Sb^vii^	136.65 (2)	Ti^vi^—Sb—Ti	113.06 (3)
Ge^iii^—Ti—Sb^vii^	136.65 (2)	Ti^vii^—Sb—Ti	113.06 (3)
Ge—Ti—Sb^vii^	81.574 (14)	Ti^iv^—Sb—Sb^v^	167.11 (4)
Sb^iv^—Ti—Sb^vii^	81.17 (2)	Ti^v^—Sb—Sb^v^	59.01 (3)
Sb^v^—Ti—Sb^vii^	81.17 (2)	Ti^vi^—Sb—Sb^v^	103.295 (14)
Sb^vi^—Ti—Sb^vii^	133.88 (5)	Ti^vii^—Sb—Sb^v^	103.295 (14)
Ge^i^—Ti—Sb	137.823 (19)	Ti—Sb—Sb^v^	54.051 (16)
Ge^ii^—Ti—Sb	137.823 (19)	Ti^iv^—Sb—Sb^iv^	59.01 (3)
Ge^iii^—Ti—Sb	137.823 (19)	Ti^v^—Sb—Sb^iv^	167.11 (4)
Ge—Ti—Sb	137.823 (19)	Ti^vi^—Sb—Sb^iv^	103.295 (14)
Sb^iv^—Ti—Sb	66.94 (3)	Ti^vii^—Sb—Sb^iv^	103.295 (14)
Sb^v^—Ti—Sb	66.94 (3)	Ti—Sb—Sb^iv^	54.051 (16)
Sb^vi^—Ti—Sb	66.94 (3)	Sb^v^—Sb—Sb^iv^	108.10 (3)
Sb^vii^—Ti—Sb	66.94 (3)	Ti^iv^—Sb—Sb^vii^	103.295 (14)
Ge^viii^—Ge—Ge^i^	180.0	Ti^v^—Sb—Sb^vii^	103.295 (14)
Ge^viii^—Ge—Ge^iii^	90.0	Ti^vi^—Sb—Sb^vii^	167.11 (4)
Ge^i^—Ge—Ge^iii^	90.0	Ti^vii^—Sb—Sb^vii^	59.01 (3)
Ge^viii^—Ge—Ge^ix^	90.0	Ti—Sb—Sb^vii^	54.051 (16)
Ge^i^—Ge—Ge^ix^	90.0	Sb^v^—Sb—Sb^vii^	69.840 (16)
Ge^iii^—Ge—Ge^ix^	180.0	Sb^iv^—Sb—Sb^vii^	69.840 (16)
Ge^viii^—Ge—Ti^i^	118.344 (11)	Ti^iv^—Sb—Sb^vi^	103.295 (14)
Ge^i^—Ge—Ti^i^	61.656 (11)	Ti^v^—Sb—Sb^vi^	103.295 (14)
Ge^iii^—Ge—Ti^i^	118.344 (11)	Ti^vi^—Sb—Sb^vi^	59.01 (3)
Ge^ix^—Ge—Ti^i^	61.656 (11)	Ti^vii^—Sb—Sb^vi^	167.11 (4)
Ge^viii^—Ge—Ti^x^	61.656 (11)	Ti—Sb—Sb^vi^	54.051 (16)
Ge^i^—Ge—Ti^x^	118.344 (11)	Sb^v^—Sb—Sb^vi^	69.840 (16)
Ge^iii^—Ge—Ti^x^	118.344 (11)	Sb^iv^—Sb—Sb^vi^	69.840 (16)
Ge^ix^—Ge—Ti^x^	61.656 (11)	Sb^vii^—Sb—Sb^vi^	108.10 (3)

Symmetry codes: (i) −*x*+1, −*y*, −*z*; (ii) *x*−1, *y*, *z*; (iii) −*x*+1, −*y*+1, −*z*; (iv) −*x*+1, −*y*+1, −*z*+1; (v) −*x*, −*y*, −*z*+1; (vi) −*x*, −*y*+1, −*z*+1; (vii) −*x*+1, −*y*, −*z*+1; (viii) −*x*+2, −*y*+1, −*z*; (ix) −*x*+2, −*y*, −*z*; (x) *x*+1, *y*, *z*.
